# Supplementary material for: Hydrogel-Based Microneedle as a Drug Delivery System
Source: Pharmaceutics. 2023 Oct 10;15(10):2444. doi: 10.3390/pharmaceutics15102444 (PMC10609870; doi:10.3390/pharmaceutics15102444)
Supplement: Supplementary file 1 [file pharmaceutics-15-02444-s001.zip › pharmaceutics-2602094-supplementary.pdf]

Table S1: Supporting data for "New approaches for hydrogel-based MN patch" topic.

**4.1.1 STIMULUS RESPONSE**				
PVA with S-nitrosoglutathione and graphene oxide (GO)	Nitric oxide (NO) **DSP:** biofilm-infected chronic wounds	Forming MN	The test results showed that an increase in temperature increased the release of NO from the patch and thus enhanced antibacterial performance.	[67]
pNIPAM	Insulin **DSP:** No specified	Hollow MNs	The system exhibited an evident volume shrinkage, excellent photothermal ability and repeatable NIR-responsiveness.	[110]
Polyvinylpyrrolidone MNs coated with chitosan and Poly (vinyl alcohol) Hydrogel	Doxorubicin and AuMSS nanorods (Dox@MicroN) **DSP:** Cervical Cancer	Coated MN	MNs structures can efficiently penetrate the tumor-mimicking agarose gel and release the Dox with a pH- and thermo-responsive profile. Furthermore, the system composed of Dox and AuMSS nanorods was able to simultaneously mediate the chemo- and photothermal-therapies rendered a superior cytotoxic effect against the cervical cancer cells	[15]
Mussel adhesive protein (MAP)-based shell and a non-swellable silk fibroin (SF)-based core	Fluorescein isothiocyanate (FITC)-conjugated dextran **DSP:** Wound Healing	Forming MN	The protein-based hydrogel system achieved *in vitro* sustained releases for at least 7 days via swelling-mediated diffusion and enzymatic degradation	[45]
Chitosan-porous carbon nanocomposites	Cephalexin **DSP:** No specified	Dissolving MNs	The system responded to acidic pH 4 and electric pulse 5 V for drug release	[112]
Methacrylated Hyaluronic Acid	Estrogen receptor alpha (ERα)-degrading PROTAC - ERD308 and Palbociclib DSP: ER-positive breast cancer	Disolving MNs	The patch was effective in overcoming the problems involved with PROTAC systems, which had high rates of local drug retention (87%). Furthermore, the combination with pH-responsive micelles allowed the co-release of Palbociclib specific to the tumor acid environment for effective therapy against breast cancer.	[27]
**4.1.2 INDUCED CONTROLLED RELEASE**				
PVA and poly(ethyl-eneimine) with MBA how cross-linking agent	Indomethacin **DSP:** Anti-inflammatory effect	Dissolving MNs	This investigation provided the efficacy of the device for attaining electro-modulated drug release in the ex vivo porcine model.	[78]
PVA with poly(ethyleneimine) solution (PEI) and 1 vinylimidazole (VI)	Indomethacin **DSP:** Anti-inflammatory effect	Dissolving MNs	Both *in vitro* and *in vivo* studies revealed a good preliminary indication that the system have electro-responsive capabilities, ultimately facilitating the immediate release of indomethacin.	[84]
Polyvinylpyrrolidone based graphene oxide HG	L-Ascorbic acid **DSP:** No specified	Dissolving MNs	New platform electricity responsive for drug deliver. The drug-release efficiency of the system with graphene oxide was increased by more than 2 times compared to the system without graphene oxide when 5 V was applied.	[95]
PVP with gold and silver electrodes	Ascorbic Acid **DSP:** No specified	Hydrogel Dissolving MN	The system was extremely efficient in controlling drug release. It was influenced by temperature, irradiation and electricity.	[98]

**4.1.3 SUSTAINED AND LONG-TERM THERAPY**				
Gelatin methacryloyl (GelMA)	Doxorrubicin<br>**DSP:** Melanoma	Dissolving MNs	The drug release rate can be adjusted by controlling the degree of polymer cross-linking.	[10]
Poly (vinyl alcohol)	Doxorubicin<br>**DSP:** Skin cancer	Forming MNs	The insertion of MNs resulted in a significantly greater delivery of doxorubicin into and across human skin, as compared to passive diffusion	[17]
β-Cyclodextrin Conjugated Gelatin Methacryloyl	Curcumin<br>**DSP:** Melanoma	Dissolving MNs	The Gelatin methacryloyl (GelMA) -β-CD/CUR MN exhibits relatively higher therapeutic efficacy through more localized and deeper penetrated manner compared with a control nontransdermal patch for anticancer activities. *In vivo* studies also verify biocompatibility and degradability of the Gelatin methacryloyl (GelMA) -β-CD MN arrays patch.	[9]
Sodium alginate and sulfobetaine methacrylate using *N, N′*-methylenebisacrylamide and Ca2+	Doxorrubicin and Lipopolissacarides<br>**DSP:** Glioma Tumor	Dissolving MNs	Adequate amounts of drugs were released from the MNs in the first 24 h, followed by a very gradual and sustained release for the next 7 d.	[20]
PVA	Rhodamine B<br>**DSP:** No specified	Dissolving MN	In this study, the system showed dissolution after 17 minutes and good swelling properties and a consistent release of the model drug RD up to 12 days	[100]
Gelatin Methacryloyl	Galunisertib<br>**DSP:** Myocadiac infarction	Dissolving MNs	The drug-release properties can be controlled by adjusting the degree of crosslinking to ensure sustained release for at least 15 days after application.	[7]
Polyethylene glycol diacrylate (PEGDA)	Gencitabine<br>**DSP:** Inflammatory breast cancer	Coated MNs	Thinking about a rapid release system, 100% of the drug was released in the first hour of administration with a system manufactured with high retention.	[25]
Multifunctional Silk Fibroin Methacryloyl	Prussian blue nanozymes (PBNs) and vascular endothelial growth factor (VEGF)<br>**DSP:** Diabetic Wound Healing	Dissolving MNs	The system exhibits excellent biocompatibility, drug-sustained release, pro angiogenesis, antioxidant, and antibacterial properties with sustained release after 9 days.	[54]
Ti_2C_3 MXenes-integrated poly-γ-glutamic acid (γ-PGA) hydrogel MNs	Asiaticoside<br>**DSP:** Diabetic Foot Ulcer	Dissolving MNs	The system was shown to be a multifunctional subcutaneous drug-delivering system for accelerating diabetic wound healing for 14 days.	[55]
Polyvinyl Alcohol	Parathyroid hormone (PTH)<br>**DSP:** Wound Healing	Forming MNs	Was demonstrated an intermittent systemic administration of PTH using our PTMN patches accelerated skin wound healing, evaluated for 14 days	[41]
Poly (vinyl alcohol) and poly (methyl vinyl ether co-maleic anhydride/acid (Gantrez® S-97), polyethylene glycol (PEG 10,000) and Na_2CO_3.	Bevacizumab<br>**DSP:** Immunotherapy for cancer	Dissolving and Forming MN	BEV was detected and measured in plasma across 7 days following one single application of MN arrays. BEV had lymphatic accumulation *in vivo*. This could prove to be a viable option for treatment of lymphomas and secondary metastatic tumors.	[18]

4.1.4 LOCALIZED AND SYSTEMIC TREATMENT				
Gantrez® S-97and PEG	Esketamine (ESK) **DSP:** Depression	Forming MNs	The authors aimed to achieve sustained therapeutic levels in plasma over 24 h using ESK-containing drug reservoirs in combination with hydrogel forming MNs in this *in vivo* feasibility study.	[58]
Poly(vinylpyrrolidone) or Gantrez® S-97	Donepezil **DSP:** Alzheimer	Forming MN	The authors aimed to achieve sustained therapeutic levels in plasma over 24 h, using the optimum patch formulation.	[59]
Poly-c-glutamic acid (c-PGA) and poly vinyl pyrrolidone-polyvinyl alcohol (PVP/PVA)	Insulin **DSP:** Diabetes	Dissolving MNs	The MNs patch after insertion, dissolve into the skin within 4 min to deliver the entire drug load, without requiring the users to remove any sharps or waste.	[49]
Carboxymethyl cellulose	Protein MERS-S1f, MERS-S1fRS09, MERS-S1ffliC, SARS-CoV-2-S1, or SARSCoV-2-S1fRS09 for antigen stimulator **DSP:** Corona Virus (SARS-CoV-2)	Dissolving MNs	The Hydrogel-based vaccine MNs elicited strong and long-lasting antigen-specific antibody responses	[28]
Gantrez® S-97 and poly(ethylene glIcol) (PEG)	Protein antigen ovalbumin (OVA) **DSP:** Adjuvant for vaccination	Forming and Dissolving MN	The system has yielded enhanced immune responses, suggesting the possibility lower dosing and equivalent outcomes as traditional needle and syringe methods.	[29]
PVA and polyvinyl pyrrolidone (PVP)	Sildenafil citrate **DSP:** Erectly Disfunction	Forming MN	The ex vivo permeation study showed that up to 80% of Sildenafil Citrate was delivered transdermally from this combined dosage in evaluation of 14 days	[76]
PVA and polyvinyl pyrrolidone (PVP)	Sildenafil citrate **DSP:** Pulmonary hypertension	Forming MN	Increasing SC bioavailability in treating pulmonary arterial hypertension is another benefit of this preparation	[93]

**4.1.5 SELF ACTIVE**				
Chitosan polyethylenimine antimicrobial Polymer	Amphotericin B<br>**DSP:** Fungal Infections	Dissolving MN	The results of combination system attributable to the high bioavailability of therapeutics and synergistic actions of the antifungal polymer and drug.	[72]
Chitosan-NIPAM Hydrogel	Vascular endothelial growth factor (VEGF)<br>**DSP:** Precaution of infections and promotion of Wound Healing	Dissolving MNs	The system exhibited a superior capability of acceleration in inflammatory inhibition, collagen deposition, angiogenesis, and granulation tissue formation. In addition, a controlled release can be achieved with thermoresponsive NIPAM	[70]
Chitosan (CS) and zinc nitrate (CS-Zn [II] MNs)	Chitosan and Zn NP<br>**DSP:** Bacterial Biofilm	Dissolving MNs	The system which not only can directly transport CS and Zn^{2+} into the bacterial biofilm, but also offer a large specific surface area, to facilitate the diffusion within biofilm and effectively eradicate the bacterial biofilm.	[71]
**4.1.6 PRECAUTION OF SIDE EFFECTS AND LOW BIOAVAILABLE**				
Photocrosslinked methacrylated hyaluronic acid (MeHA)	Glucagon<br>**DSP:** Diabetes treatment problems	Dissolving MN	The system successfully prevented hypoglycemia induced by overdosed insulin administration in rat model. The results from this work warrants further development of the transdermal glucagon delivery system as a solution to potential lifethreatening complications associated with intensive insulin therapy	[52]
Polyacrylic acid	Naloxone<br>**DSP:** Management of side effects of opioids	Dissolving MNs	Naloxone permeation through intact skin was highest from pH 7.4 gels when naloxone is unionized	[92]
Gelatin methacryloyl (GelMA)	L-DOPA<br>**DSP:** Parkinson	Dissolving MNs	The system has reached a released L-DOPA directly entered the blood, which reduces the side effects on the gastrointestinal tract and improves the utilization rate of the drug.	[60]
